# Effects of COVID-19 Acute Respiratory Distress Syndrome Intensive Care Unit Survivor Telemedicine Clinic on Patient Readmission, Pain Perception, and Self-Assessed Health Scores: Randomized, Prospective, Single-Center, Exploratory Study

**DOI:** 10.2196/43759

**Published:** 2023-03-22

**Authors:** Bathmapriya Balakrishnan, Lucas Hamrick, Ariful Alam, Jesse Thompson

**Affiliations:** 1 Section of Pulmonary, Critical Care and Sleep Medicine Department of Medicine West Virginia University Morgantown, WV United States; 2 Pulmonary and Critical Care Medicine Institute for Academic Medicine Charleston Area Medical Center Charleston, WV United States; 3 Department of Medicine West Virginia University Morgantown, WV United States

**Keywords:** acute respiratory distress syndrome, aftercare, COVID-19 pneumonia, critical care, survivor, telemedicine

## Abstract

**Background:**

Post-intensive care syndrome (PICS) affects up to 50% of intensive care unit (ICU) survivors, leading to long-term neurocognitive, psychosocial, and physical impairments. Approximately 80% of COVID-19 pneumonia ICU patients are at elevated risk for developing acute respiratory distress syndrome (ARDS). Survivors of COVID-19 ARDS are at high risk of unanticipated health care utilization postdischarge. This patient group commonly has increased readmission rates, long-term decreased mobility, and poorer outcomes. Most multidisciplinary post-ICU clinics for ICU survivors are in large urban academic medical centers providing in-person consultation. Data are lacking on the feasibility of providing telemedicine post-ICU care for COVID-19 ARDS survivors.

**Objective:**

We explored the feasibility of instituting a COVID-19 ARDS ICU survivor telemedicine clinic and examined its effect on health care utilization post-hospital discharge.

**Methods:**

This randomized, unblinded, single-center, parallel-group, exploratory study was conducted at a rural, academic medical center. Study group (SG) participants underwent a telemedicine visit within 14 days of discharge, during which a 6-minute walk test (6MWT), EuroQoL 5-Dimension (EQ-5D) questionnaire, and vital signs logs were reviewed by an intensivist. Additional appointments were arranged as needed based on the outcome of this review and tests. The control group (CG) underwent a telemedicine visit within 6 weeks of discharge and completed the EQ-5D questionnaire; additional care was provided as needed based on findings in this telemedicine visit.

**Results:**

Both SG (n=20) and CG (n=20) participants had similar baseline characteristics and dropout rate (10%). Among SG participants, 72% (13/18) agreed to pulmonary clinic follow-up, compared with 50% (9/18) of CG participants (*P*=.31). Unanticipated visits to the emergency department occurred for 11% (2/18) of the SG compared with 6% (1/18) of the CG (>.99). The rate of pain or discomfort was 67% (12/18) in the SG compared with 61% (11/18) in the CG (*P*=.72). The anxiety or depression rate was 72% (13/18) in the SG versus 61% (11/18; *P*=.59) in the CG. Participants’ mean self-assessed health rating scores were 73.9 (SD 16.1) in the SG compared with 70.6 (SD 20.9) in the CG (*P*=.59). Both primary care physicians (PCPs) and participants in the SG perceived the telemedicine clinic as a favorable model for postdischarge critical illness follow-up in an open-ended questionnaire regarding care.

**Conclusions:**

This exploratory study found no statistically significant results in reducing health care utilization postdischarge and health-related quality of life. However, PCPs and patients perceived telemedicine as a feasible and favorable model for postdischarge care among COVID-19 ICU survivors to facilitate expedited subspecialty assessment, decrease unanticipated postdischarge health care utilization, and reduce PICS. Further investigation is warranted to determine the feasibility of incorporating telemedicine-based post-hospitalization follow-up for all medical ICU survivors that may show improvement in health care utilization in a larger population.

## Introduction

In the United States, 5 million patients are admitted to the intensive care unit (ICU) every year [1]. Post-intensive care syndrome (PICS) affects up to 50% of ICU survivors, leading to new long-term neurocognitive, psychosocial, and physical impairments [2]. Approximately one-third of ICU survivors do not return to work, while another one-third of patients do not go back to their pre-ICU job or receive a salary at a similar level to their pre-ICU state [3]. The scale and impact of critical illnesses requiring an ICU stay are staggering.

After hospital discharge, ICU survivors were not more likely to be rehospitalized within 30 days compared with non-ICU hospitalized patients (15.8% vs 16.1%, *P*=.08) [4]. However, they were more likely to receive post-acute care services (45.3% vs 70.9%, *P*<.001) [4]. Readmission among patients with severe sepsis, the most common admission diagnosis in the medical ICU, exceeded 40% in the first 3 months after hospital discharge; of these cases, 42% were found to be potentially preventable [5]. These data suggest that effective and timely postdischarge follow-up is paramount to addressing the high morbidity among ICU survivors and reducing the readmission rates.

Regardless of their severity of illness, most patients discharged from the hospital are seen in transitional care clinics administered by the admitting hospital or by the patient’s primary care physician (PCP) within 7 days to 14 days [6]. These care models do not directly address an ICU survivor's complex health care needs. The Society of Critical Care Medicine advocates for the assessment of ICU survivors for physical, cognitive, and emotional problems after hospital discharge [7]. ICU recovery programs have been implemented over the last 25 years in Europe. However, there are scant and inconsistent data on the ideal care delivery model, subspecialty participation, and evidence demonstrating benefit in health-related quality of life (HRQoL) measures [8]. Despite a wide variation in the composition, size, and care delivery model of each post-ICU clinic, a multidisciplinary, team-based approach is core to the functioning of these clinics [9]. These programs, additionally, have an excessive no-show rate of up to 36%, a majority of which is due to nonattendance and appointment cancellations [10]. Thus, more prospective randomized studies should be implemented to determine the best method to deliver care with a lower attrition rate.

More than 19.3% of the US population live outside of urban areas [11], and these rural populations have additional barriers to health care, including higher uninsured and poverty rates, lower literacy, lack of transportation, functional disability, financial burden, and poor access roads [12,13]. West Virginia (WV) is the third most rural state in the nation, with 51.8% of residents living in rural areas. There are higher rates of chronic diseases and a scarcity of physicians among socioeconomically disadvantaged rural populations in WV compared with the rest of the nation [12].

Acute respiratory distress syndrome (ARDS) affects up to 42% of patients with COVID-19 pneumonia, and in the ICU, the rates are higher; between 61% and 81% of ICU patients with COVID-19 pneumonia develop ARDS [13]. The 2012 Berlin Criteria for ARDS include the presence of (1) acute hypoxemic respiratory failure, (2) presentation within 1 week of respiratory symptom development, (3) bilateral airspace disease on chest imaging, and (4) the exclusion of heart failure as the primary cause of acute hypoxemic respiratory failure [14]. Despite the prevalence of ARDS in patients with COVID-19, post-ICU clinic outcomes have not been well researched.

Due to the COVID-19 pandemic, utilization of telemedicine exponentially increased 63-fold from 2019 to 2020 [15]. Telemedicine can deliver effective health care in an organized manner at the convenience of patients and medical providers, especially in remote areas where primary care and subspecialty care are scarce [16]. Technological advances and the recent pandemic have fueled the paradigm shift in the acceptability of remote screening, assessment, and management of patients. However, the implementation of telemedicine for postdischarge management of ICU survivors has not been studied.

We conducted an exploratory study comparing a COVID-19 ARDS ICU survivor telemedicine clinic to usual care at a rural academic medical center. This study is innovative because it explores the potential of a novel health care model, namely time-sensitive telemedicine follow-up for the transitional care of rural adult ICU survivors. We hypothesized that this clinical program would increase follow-up in the pulmonary clinic and decrease unanticipated health care utilization within 30 days of hospital discharge. Secondarily, we hypothesized that this program would improve HRQoL measures.

## Methods

### Participants and Study Sites

Between July 2021 and December 2021, all adults (≥18 years of age) admitted to the medical ICU with the diagnosis of ARDS from COVID-19 pneumonia and discharged home were approached for recruitment in the study. Exclusion criteria included (1) lack of communication and internet services, (2) pre-admission medical comorbidities preventing independent self-care, (3) discharge to hospice care or a long-term acute care hospital, (4) substance abuse or psychiatric disorder that prevents independent living, (5) patients who were blind or deaf, (6) non-English speakers, (7) chronic ventilator use prior to ICU hospitalization, (8) nursing home or rehabilitation residence prior to ICU hospitalization, and (9) solid or hematopoietic transplant patients.

### Randomization

Enrollment continued until each group had 20 patients. Participants were randomized to the study group (SG) or control group (CG) in a 1:1 ratio by computer-generated simple randomization without stratification. Assignments remained concealed to study personnel until patients met the inclusion and exclusion criteria, consented, enrolled, and were entered into the study database. The SG participants were provided with a calibrated portable pulse oximeter and an automated blood pressure (BP) monitor.

### Study Group Interventions

The SG participants underwent a telemedicine visit within 14 days of discharge, at which daily vital signs logs (VSL) were reviewed and the 6-minute walk test (6MWT) and EuroQoL 5-Dimension (EQ-5D) questionnaire were completed. The telemedicine visit was conducted by a dual-trained intensivist and pulmonologist (BB). Telemedicine visits were 30 minutes to 40 minutes long, resembling a pulmonary outpatient clinic assessment, in addition to completion of the study protocol. A standardized evaluation script was used to aid in the identification of a pulmonary problem list and to develop a treatment plan. Pulmonary medication reconciliation and counseling were provided. Finally, a targeted case management assessment was provided with durable medical equipment and supplemental oxygen prescriptions, referral to outpatient physical therapy, home health, and PCPs, as needed.

### Control Group Interventions

#### Overview

The CG underwent a telemedicine visit within 6 weeks of discharge and completed the EQ-5D questionnaire. A telemedicine visit was scheduled at 6 weeks because CG participants were seen by their PCPs or the transitional care clinic at West Virginia University (WVU) within 7 days to 14 days of hospital discharge. The telemedicine visit was conducted by BB. Telemedicine visits were 20 minutes to 30 minutes long, resembling a pulmonary outpatient clinic assessment. A standardized evaluation script was used to identify a pulmonary problem list and to develop a treatment plan. Pulmonary medication reconciliation and counseling were provided. Finally, a targeted case management assessment was completed.

The study protocol is summarized in Table 1. All participants in the SG and CG were offered an in-person pulmonary clinic follow-up at the conclusion of the telemedicine encounter. All telemedicine encounters were conducted from a private room at the Physician Office Center, Ruby Memorial Hospital, WVU. Secure telephone and internet connections were used to complete the telemedicine encounter with the study participants. All documentation was completed in the EPIC electronic hospital record.

**Table 1:** Study protocol.

Study protocol		Control group (n=20)	Study group (n=20)
**Telemedicine visit—within 14 days of hospital discharge**			
	Daily vital sign log review	No	Yes
	6-minute walk test	No	Yes
	EQ-5D^a^ questionnaire	No	Yes
	Pulmonary problem list evaluation	No	Yes
	Pulmonary medication reconciliation	No	Yes
	Treatment plan	No	Yes
	Counseling	No	Yes
**Telemedicine visit—within 6 weeks of hospital discharge**			
	EQ-5D questionnaire	Yes	No
	Pulmonary problem list evaluation	Yes	No
	Pulmonary medication reconciliation	Yes	No
	Treatment plan	Yes	No
	Counseling	Yes	No
Poststudy feedback — within 4 weeks of completion of study		Yes	Yes

^a^EQ-5D: EuroQoL 5-Dimension; a standardized measure of health-related quality of life.

#### Daily Vital Signs Log (VSL)

Participants were required to record their daily BP, heart rate, and pulse oximetry at rest. Participants were advised to obtain these measurements at a consistent time each day. Heart rate and pulse oximetry were recorded immediately after exertion. Additionally, the amount of oxygen supplementation used, if any, was recorded in liters per minute (L/min). Participants recorded their daily VSL from day 1 to day 14 postdischarge from the hospital.

#### EuroQoL 5-Dimension Questionnaire

The EQ-5D tool is an HRQoL questionnaire based on each rater’s self-assessment scores. “The scale measures quality of life on a 5-component scale including mobility, self-care, usual activities, pain/discomfort, and anxiety/depression. Each level is rated on [a] scale that describes its degree of problems in that area” [17]. The questionnaire also invites the participant to give an overall health rating by choosing a number between 0 and 100; the chosen rating represents the person’s perceived state of health that day from “worst imaginable” to “best imaginable.” The EQ-5D questionnaire is a reliable tool with an average test-retest reliability using interclass coefficients with means of 0.78 and 0.73 [17].

#### 6-Minute Walk Test

The 6MWT is a functional capacity assessment method for patients with cardiopulmonary conditions usually performed in a pulmonary function laboratory. Mak et al [18] used iPhones and Apple Watches to measure the reliability of the 6MWT performed outside the laboratory. For patients performing an in-clinic 6MWT, the researchers noted an error between ground truth and phone-calculated steps ranging from 2.0% to 8.0% [18]. Our study used the 6MWT as a surrogate marker of functional impairment following hospitalization. During their inpatient stay, participants in the SG were given instructions on how to install the iWalkAssess mobile app on their smartphones. A supervised test was performed during their inpatient stay to provide guidance on how to conduct this test at home. SG participants were asked to perform and record their total distance walked in meters on day 14 prior to their telemedicine encounter.

### Poststudy Feedback From Patients and PCPs

A poststudy survey was administered by BB and AA to study participants and PCPs 4 weeks after the completion of the study in February 2022 (Textbox 1). All surveys were completed via telephone calls to study participants. Up to 3 attempts to contact study participants were made, after which they were deemed unreachable. PCPs were contacted via telephone and email up to 3 times to complete the poststudy survey. If no responses were received after 1 week, they were deemed unreachable.

**Poststudy interview questions to patients and primary care physicians (PCPs).**

**Patients**

1. Do you recall your telemedicine visit?
2. Did you find the telemedicine visit to be useful?
3. Did the telemedicine clinic help you with setting up follow-up appointments & tests?
4. Were the blood pressure (BP) cuff and pulse oximeter useful to you?
5. Are you using the BP cuff and pulse oximeter now?
6. Were you able to come off oxygen? (if patient was discharged with home oxygen after COVID-19 hospitalization)
7. Do you think it will be a good idea to do follow-up telephone visits by an intensive care unit (ICU) physician & team?

**PCP**

1. Did you receive documentation of the telemedicine encounter?
2. Did you find the telemedicine clinic encounter useful?
3. Did it improve continuity of care from ICU hospitalization?
4. Was it useful in clarifying management of diagnoses post ICU hospitalization?
5. Was it useful in oxygen weaning?
6. Did it help you/your patients with establishing follow-up with subspecialty clinics?

### Outcomes

The primary outcome of this exploratory study was health care utilization by participants within 30 days of hospital discharge. We determined participants’ agreement to attend an in-person pulmonary clinic, attendance at the pulmonary clinic, PCP follow-up, and unanticipated visits to the emergency department (ED) or urgent care (UrC). Unanticipated health care utilization in the ED or UrC were defined as unplanned visits to these facilities by study participants for reasons that do not relate to their index hospitalization and ICU stay for COVID-19 ARDS as determined by the study investigators. The secondary outcome of the study was HRQoL measurements. Other endpoints included the proportion of participants who were weaned off oxygen supplementation, compliance to self-monitoring using the daily VSL, 6MWT, and, finally, perception of PCPs and participants regarding this novel program.

### Data Collection

Data on demographics, severity of illness at hospital and ICU admission, treatment for COVID-19 ARDS, length of stay (LOS) in the hospital and the ICU, pregnancy status, and discharge needs were recorded. Inpatient diagnoses were manually extracted from the electronic health record. The authors (BB and AA) interviewed participants and their respective PCPs via telephone 4 weeks after the completion of the study to gain the respondents’ subjective impressions of the study. See the interview protocol (Textbox 1) for a description of the interview questions.

### Statistical Methods

This pilot study had the institutional funding to support enrollment of 40 participants over the course of 6 months. Data analyses were conducted in R. Means and standard deviations or medians and interquartile ranges were calculated for each outcome at baseline and postintervention. When feasible, statistical significance was determined by using a Chi-square test, Fisher exact test, *t* test, or Wilcoxon rank sum test to compare the SG and CG outcomes. Content analysis of participants’ responses identified the themes of intervention acceptability. Additionally, PCPs were interviewed to examine the ease and utility of the intervention.

### Ethical Considerations

This was a prospective, randomized, unblinded, single-center, parallel-group, exploratory study. The institutional review board (IRB) at WVU approved the study (#2104284924) on July 7, 2021. The purpose, risks, and benefits of the study were discussed with all participants prior to obtaining their informed consent. Additionally, the informed consent contained language indicating that participants would not be compensated for participation, willingness to participate would not affect their quality of care, data gathered as a participant could be used for research purposes, and that all data would be presented deidentified and in aggregate. The study was conducted in accordance with the ethical standards of the WVU IRB on human experimentation and with the Helsinki Declaration of 1975. All study records were securely stored in the REDCap system at WVU in accordance with WVU Health Sciences Center data security policies for protected health information.

### Consent for Publication

Each patient or closest relative was informed of the anonymous prospective collection of data and had the option of choosing not to participate in the study. Accordingly, in case of refusal, the data were not collected.

## Results

### Recruitment and Retention

**Figure 1:**
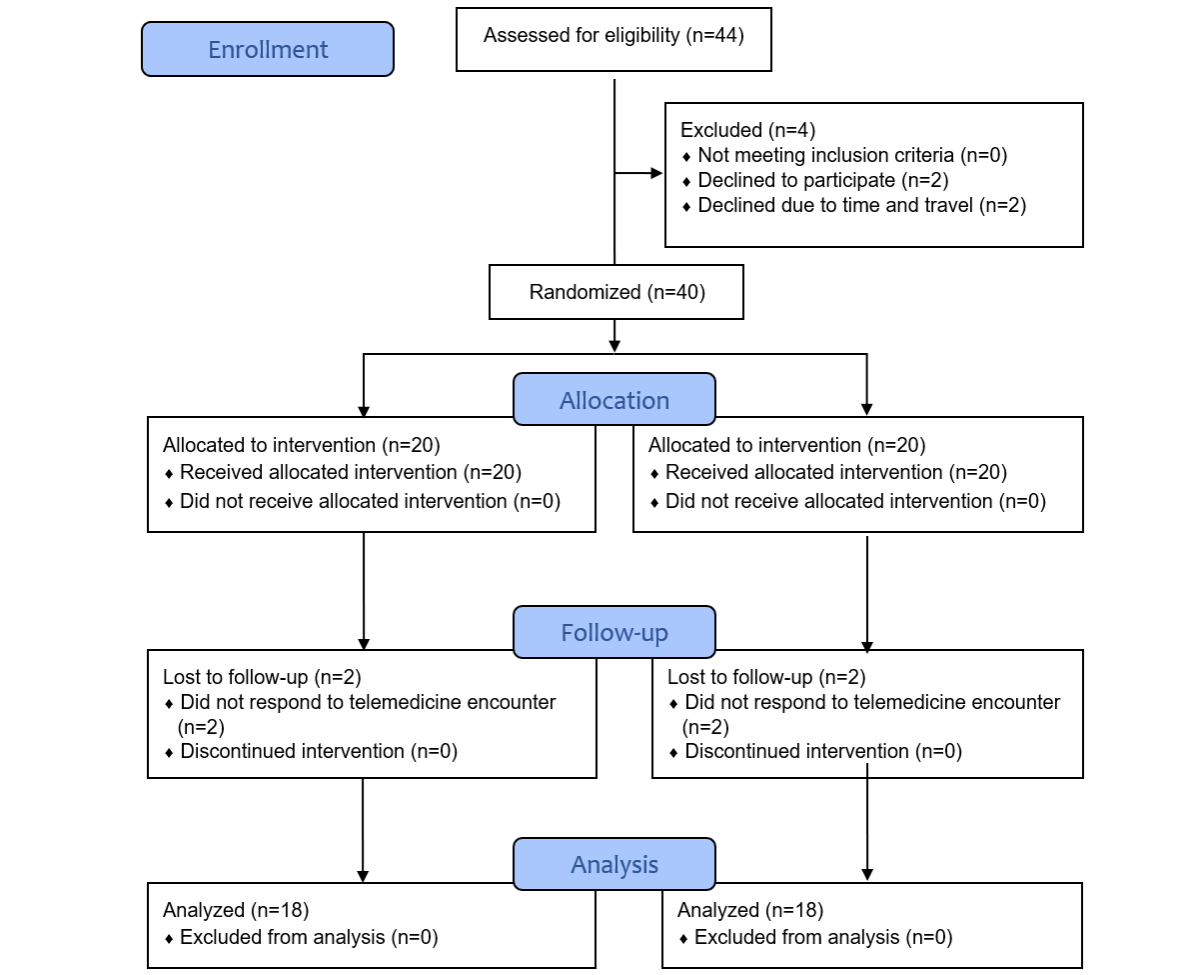
CONSORT (Consolidated Standards of Reporting Trials) flow diagram illustrating study recruitment.

During the study period, 44 participants met the eligibility criteria. Of the 4 participants who declined involvement in the study, 2 declined to participate after learning more about the demands of the program, and the remaining 2 cited time and travel constraints. We enrolled and randomized 40 participants to either the SG or CG (Figure 1). Recruitment ended after each group had 20 participants. Of the participants in the SG and CG, 90% (18/20 each) completed all components of the study. There was a 10% drop-out rate in both groups. All 4 patients who dropped out were lost to follow-up despite more than 5 attempts over 2 days to complete the telemedicine encounter. Recruitment began in July 2021, and the study was completed by January 2022. A poststudy survey was administered to participants and PCPs 4 weeks after the completion of the study in February 2022.

### Baseline Data

The baseline characteristics of participants in both the SG and CG are shown in Table 2. Both groups had a broadly similar demographic profile, severity of illness, oxygen delivery method in the ICU, LOS in the hospital and ICU, pregnancy status, and discharge needs.

**Table 2:** Baseline characteristics of participants.

Demographics			Control group (n=20)		Study group (n=20)		*P* value	
Male, n (%)			12 (60)		9 (45)		.53^a^	
Age (years), mean (SD)			50.20 (11.50)		47.00 (13.88)		.43^b^	
BMI (kg/m^2^), mean (SD)			32.75 (8.67)		37.94 (8.15)		.058^b^	
Completed the study, n (%)			18 (90)		18 (90)		>.99^b^	
Comorbidities, median (IQR)			2.5 (1.00-4.25)		3.00 (2.00-5.50)		.55^b^	
**Smoking history, n (%)**								
	Ex-smoker	8 (40)		4 (20)		.30^a^		
	Current smoker	2 (10)		1 (5)		>.99^b^		
	e-cigarette user	0		0		>.99^b^		
Pregnant, n (%)			1 (5)		3 (15)		>.99^b^	
Gestation (weeks), mean (SD)			31.00 (N/A)		26.00 (4.00)		>.99^b^	
**WHO^c^ severity at hospital admission, n (%)**								.40^b^
	Moderate	6 (30)		2 (10)				
	Severe	9 (45)		12 (60)				
	Critical	5 (25)		6 (30)				
**WHO severity at ICU^d^ admission**								>.99^b^
	Moderate	3 (15)		2 (10)				
	Severe	6 (30)		6 (30)				
	Critical	11 (55)		12 (60)				
mSOFA^e^ score Day 1 ICU stay, median (IQR)			4.0 (3-6.5)		4 (4-5.25)		.86^b^	
HFNC^f^ (days), median (IQR)			0.00 (0-3.25)		1.00 (0-2.00)		.50^b^	
NIV^g^ (days), median (IQR)			1.00 (0-2.25)		1.00 (0-5.25)		.54^b^	
MV^h^ (days), median (IQR)			1 (0-7)		0 (0-3.75)		.68^b^	
**Medication, n (%)**								
	Tocilizumab	2 (10)		2 (10)		>.99^b^		
	Remdesivir	1 (5)		5 (25)		.18^i^		
	Dexamethasone	17 (85)		19 (95)		.61^i^		
	Baricitinib	9 (45)		8 (40)		>.99^b^		
	IVIG^j^	0		1 (5)		>.99^b^		
Paralysis, n (%)			5 (25)		3 (15)		.70^b^	
Proning, n (%)			5 (25)		3 (15)		.45^b^	
Length of hospitalization (days), median (IQR)			11.50 (7-16.25)		12 (9.75-18)		.22^b^	
Length of ICU stay (days), median (IQR)			5.5 (2.75-9.25)		4.5 (4-7)		.96^b^	
Number of hospital problems at discharge, median (IQR)			3.5 (2.75-4.25)		4 (2.75-4)		.74^b^	
Baby delivered, n (%)			0 (0)		1 (5)		>.99^b^	
Pregnancy intact at discharge, n (%)			1 (5)		2 (10)		>.99^b^	
**Discharge needs, n (%)**								
	Home oxygen	14 (70)		15 (75)		>.99^b^		
	Home health	4 (20)		4 (20)		>.99^b^		
	OT^k^ or PT^l^	5 (25)		3 (15)		.70^i^		

^a^Chi-square test.

^b^*t* test.

^c^WHO: World Health Organization.

^d^ICU: intensive care unit.

^e^mSOFA: modified sequential organ failure assessment.

^f^HFNC: high-flow nasal cannula.

^g^NIV: noninvasive ventilation.

^h^MV: mechanical ventilation.

^i^Fisher test.

^j^IVIG: intravenous immunoglobulin.

^k^OT: occupational therapy.

^l^PT: physical therapy**.**

### Numbers Analyzed

A total of 36 participants completed all components of the study. Thus, they were included in the final analyses. All analyses were performed in participants’ originally assigned groups.

### Postdischarge Utilization Within 30 Days

Participants completed their telemedicine follow-up within a mean 16.7 (SD 4.5) days in the SG and 29.4 (SD 3.5) days in the CG (*P*<.001; Table 3). All (36/36, 100%) participants completed their PCP visits prior to their telemedicine visit. Compared with 72% (13/18) of SG participants, only 50% (9/18) of CG participants agreed to follow-up in the pulmonary clinic (*P*=.31). The no-show rate was 11% (2/18) in the CG compared with 6% (1/18) in the SG. The rate of unanticipated visits to the ED and UrC was 11% (2/18) in the CG compared with 6% (1/18; *P*>.99) in the SG. Reasons cited for these unanticipated visits included rectal bleeding, nasal singeing due to inadvertent sparking of an oxygen device, and hospital admission for failure of oral antifungal therapy for mucormycosis. None of these conditions were preventable by the telemedicine encounters.

**Table 3:** Postdischarge outcomes.

Outcomes		Control group (n=18)	Study group (n=18)	*P* value	
Time to visit postdischarge (days), mean (SD)		29.4 (3.5)	16.7 (4.5)	<.001^a^	
Agreed to in-person pulmonary follow-up, n (%)		9 (50)	13 (72)	.31^b^	
**Pulmonary clinic follow-up, n (%)**					.11^c^
	No show	2 (11)	1 (6)		
	Visit completed	2 (11)	9 (50)		
	Declined	8 (44)	5 (28)		
	Follow-up in the future	5 (28)	3 (17)		
PCP^d^ visit completed, n (%)		18 (100)	18 (100)	>.99^a^	
Unanticipated ED^e^/UrC^f^ visits, n (%)		2 (11)	1 (6)	>.99^a^	
**Daily vital signs logs, n (%)**					>.99^a^
	Did not do	0 (0)	4 (22)		
	Incomplete	0 (0)	6 (33)		
	Completed	0 (0)	8 (44)		
	Not applicable	18 (100)	0 (0)		
**6-minute walk test, n (%)**					>.99^a^
	Completed	0 (0)	9 (50)		
	Incomplete	0 (0)	9 (50)		
	Not applicable	18 (100)	0 (0)		
**Oxygen weaned, n (%)**					.59^c^
	No	6 (33)	7 (39)		
	Yes	6 (33)	8 (44)		
	Not applicable	6 (33)	3 (17)		

^a^*t* test.

^b^Chi-square test.

^c^Fisher test.

^d^PCP: primary care physician.

^e^ED: emergency department.

^f^UrC: urgent care.

### PICS

To study the development of PICS among COVID-19 ARDS ICU survivors who were discharged home, participants in the SG and CG were asked to complete the EQ-5D questionnaire (Table 4). In the SG, 94% (17/18) reported no anxiety or depression compared with 72% (13/18) in the CG (*P*=.18). The pain and discomfort domain had a small nonsignificant difference, with 12 participants in the SG reporting none and 11 participants in the CG reporting none. There were no differences between the groups in the other 3 domains. Participants’ self-assessed health rating score was 73.9 (SD 16.1) in the SG and 70.6 (SD 20.9) in the CG (*P*=.59).

**Table 4:** Standardized measure of health-related quality of life (EuroQoL-5D) questionnaire.

Domains		Control group (n=18)	Study group (n=18)	*P* value^a^	
**Mobility, n (%)**					>.99
	No problem	7 (39)	7 (39)		
	Some problem	11 (61)	10 (56)		
	Confined to bed	0 (0)	1 (6)		
**Self-care, n (%)**					>.99
	No problem	12 (67)	12 (67)		
	Some problem	6 (33)	5 (28)		
	Unable to wash or dress	0	1 (6)		
**Activities, n (%)**					>.99
	No problem	8 (44)	7 (39)		
	Some problem	10 (56)	10 (56)		
	Unable to perform usual activities	0 (0)	1 (6)		
**Pain and discomfort, n (%)**					.73
	None	11 (61)	12 (67)		
	Moderate	7 (39)	5 (28)		
	Extreme	0 (0)	1 (6)		
**Anxiety and depression, n (%)**					.18
	None	13 (72)	17 (94)		
	Moderate	5 (28)	1 (6)		
	Extreme	0 (0)	0 (0)		
Health rating score, mean (SD)		70.6 (20.9)	73.9 (16.1)	.60	

^a^Fisher test or *t* test.

### Compliance With Self-Monitoring

Table 3 illustrates the compliance of the SG participants in self-monitoring postdischarge. As the table summarizes, 44% (8/18) of the participants in the SG completed their daily VSL, 33% (6/18) partially completed this assignment, and 22% (4/18) did not complete it. Prior to their telemedicine visit, only 50% (9/18) of the SG participants completed their 6MWT. This intervention was not provided to the CG. The reasons cited for incomplete daily VSL and 6WMT by SG participants were lack of time, loss of monitoring sheets, and malfunctioning smartphones.

### Oxygen Supplementation Weaning

The 6MWT and daily VSL were administered to provide real-time tracking of participants’ recovery following COVID-19 ARDS. Differences in the need for oxygen at discharge complicated analysis. Approximately 8 of 15 patients in the SG were able to be weaned off oxygen. The CG only had 12 patients who needed oxygen at discharge, and 6 of them were able to be weaned off oxygen by the end of the study.

### Poststudy Feedback From Patients and PCPs

The poststudy survey among PCPs achieved a 17% (6/36) response rate. One-half (3/6) of the PCPs read and recollected the telemedicine encounter. Of these, 2 thought the telemedicine encounter helped establish continuity of care and clarified the patients’ hospital course following prolonged critical illness hospitalization. Additionally, a PCP commented that the SG intervention was useful in the weaning and discontinuation of oxygen supplementation therapy. All PCPs who recollected the telemedicine encounter (n=3) believed that the study helped to not only establish access but also expedite follow-up in the pulmonary clinic.

In addition, 6 patients (6/36, 17%) responded to the survey, 4 from the SG and 2 from the CG. Only 4 of the 6 respondents recalled the telemedicine encounter: 1 of 2 in the CG and 3 of 4 in the SG. The SG respondents reported using the BP cuff and pulse oximeter and that they were helpful, and 3 of 4 reported that they are still using these devices. All SG survey respondents reported that the pulse oximeter and automatic BP monitor were easy to use, and 75% (3/4) were still using these instruments following the completion of the study. According to the survey results, 67% (4/6) of respondents thought the monitoring devices helped them wean off oxygen supplementation. In addition, 83% (5/6) thought it was a good idea to provide follow-up visits via telemedicine and found it reassuring to be able to speak to an intensivist during this encounter.

### Harms

No adverse events were reported among the SG and CG participants in this study.

## Discussion

Post-ICU recovery clinics aim to address the substantial impact of critical illness on an ICU survivor. This exploratory study compared a post-ICU recovery telemedicine clinic follow-up within 14 days of hospital discharge to usual care among COVID-19 ARDS ICU survivors. We observed a nonstatistically significant higher compliance to subspecialty care, a lower rate of unanticipated postdischarge health care utilization, and a reduction in PICS. Overall, this innovative health care delivery model was well received by PCPs and participants.

Various post-ICU recovery clinics across the country are affected by high no-show rates [10]. Factors associated with nonattendance include rurality of patients, longer duration of ICU LOS, and mechanical ventilation [10]. The high degree of new cognitive, physical, and psychosocial impairments suffered by ICU survivors impedes patient engagement in health care. Redesign of ICU survivorship management and delivery systems is needed to engage patients in self-care and monitoring, early intervention for deteriorating health, and cost reduction [19]. The telemedicine model of the COVID-19 ICU survivor clinic achieved a 90% attendance rate. In-person ICU recovery programs achieve attendance rates between 20% [20] and 64% [10]. Telemedicine has the potential to reduce nonattendance to hospitalization aftercare in this cohort of patients.

Approximately 70% of patients completed posthospitalization visits during the pandemic, consistent with findings prior to the pandemic [21]. However, the proportion of telemedicine encounters increased exponentially to compensate for the decrease in in-person visits [22]. In the narrative review by Butzner and Cuffee [23], positive outcomes and experiences were reported regarding telehealth use in rural populations. It allows for a reduction in resource utilization, like physicians’ office space, and improves access to care. Telemedicine also allows for the provision of educational support for patients beyond hospitals and clinics and facilitates patient-clinician communication [24]. In this study, poststudy feedback among PCPs and participants reflects the consensus in the literature. Telemedicine post-ICU follow-up was favorable, convenient, and improved continuity and access to aftercare.

Survivors of severe COVID-19 pneumonia have a propensity for developing interstitial lung disease [25]. Up to 35% of patients with severe COVID-19 pneumonia have post-COVID-19 fibrosis on a chest computed tomogram 6 months later [26]. This underlies the importance of continued monitoring of COVID-19 ARDS ICU survivors and access to pulmonary care for these patients. In this study, all participants were offered pulmonary follow-up. Those in the SG were more likely to accept the referral compared with those in the CG. This trend was reflected in the attrition rate of participants in the pulmonary clinic. Prior studies of post-ICU survivors showed 53% of patients being referred to subspecialty care [20]. However, these data reflect prepandemic conditions and do not specify the diagnoses nor the type of subspecialty referral.

Participant compliance was subpar for self-monitoring to track recovery posthospitalization. Monitoring of post-ICU recovery is usually not provided to patients after discharge from the hospital. With the advent of wearable monitoring devices, one can envision the possibility of its incorporation to assist in clinical decision-making. Wearables may allow for early recognition of impairments with the potential for early intervention [19]. In a study of postsurgical pediatric patients, information derived from wearable monitoring devices assisted in triaging appropriateness for ED evaluation [27].

Our study has important limitations because it was conducted in a single center. The population comprised rural patients, limiting its generalizability. The small sample size reflected the pilot nature of the study, and the study’s emphasis was placed on the feasibility of utilizing telemedicine follow-up for survivors of critical illness. This study was implemented with limited resources during peak COVID-19 hospitalization. It was carried out by clinicians without additional assistance by research nurses or coordinators. The poststudy survey return rate was low, which may have overestimated the positive response observed with both participants and PCPs. Ideally, a larger study should be conducted with additional resources to include a wider inclusion criterion including higher-risk patients.

Given the limitations of this exploratory study, the preliminary results of the COVID-19 ICU survivor telemedicine clinic show that participation in the program holds promise as a feasible and acceptable method of postdischarge follow-up for ICU survivors. We intend to repeat the intervention by incorporating all medical ICU patients with a LOS of at least 48 hours into a larger controlled trial.

This randomized, single-center, exploratory study of a COVID-19 ARDS ICU survivor telemedicine postdischarge clinic found no statistically significant results in reducing health care utilization or HRQoL measures. However, our data suggest that a post-ICU recovery telemedicine clinic could facilitate expedited subspecialty assessment, decrease unanticipated health care utilization, and reduce PICS. It was also an acceptable health care delivery model for PCPs and ICU survivors. Further investigation is warranted to determine its feasibility on a larger scale by incorporating all medical ICU patients with an LOS of at least 48 hours into a larger controlled trial.

## Abbreviations

6MWT: 6-minute walk test
ARDS: acute respiratory distress syndrome
BP: blood pressure
CG: control group
ED: emergency department
EQ-5D: EuroQoL 5-Dimension
HRQoL: health-related quality of life
ICU: intensive care unit
IRB: institutional review board
LOS: length of stay
PCP: primary care physician
PICS: post-intensive care syndrome
SG: study group
UrC: urgent care
VSL: vital signs logs
WV: West Virginia
WVU: West Virginia University

## Data Availability

The data sets generated during the current study are available from the corresponding author upon reasonable request.
